# Ketamine treatment for depression: qualitative study exploring patient views

**DOI:** 10.1192/bjo.2020.165

**Published:** 2021-01-11

**Authors:** Sagar Jilka, Clarissa Mary Odoi, Emma Wilson, Sazan Meran, Sara Simblett, Til Wykes

**Affiliations:** Institute of Psychiatry, Psychology & Neuroscience, King's College London, UK; and South London and Maudsley NHS Foundation Trust, UK; Institute of Psychiatry, Psychology & Neuroscience, King's College London, UK; and South London and Maudsley NHS Foundation Trust, UK; Institute of Psychiatry, Psychology & Neuroscience, King's College London, UK; Institute of Psychiatry, Psychology & Neuroscience, King's College London, UK; Institute of Psychiatry, Psychology & Neuroscience, King's College London; and South London and Maudsley NHS Foundation Trust, UK; Institute of Psychiatry, Psychology & Neuroscience, King's College London, UK; and South London and Maudsley NHS Foundation Trust, UK

**Keywords:** Drugs of dependence disorders, qualitative research, depressive disorders, patients, ketamine treatment

## Abstract

**Background:**

Ketamine is a new and promising treatment for depression but comes with challenges to implement because of its potential for abuse.

**Aims:**

We sought the views of patients to inform policy and practical decisions about the clinical use of ketamine before large-scale roll-out is considered.

**Method:**

This qualitative study used three focus groups and three validation sessions from 14 patients with prior diagnoses of depression but no experience of ketamine treatment. Focus groups explored their views about clinical use of ketamine and the best way for ketamine to be administered and monitored. The qualitative data were analysed by three service-user researchers using thematic analysis.

**Results:**

Five themes were generated: changing public perceptions, risks, monitoring, privacy and data protection, and practical aspects. Participants were conscious of the stigma attached to ketamine as a street drug and wanted better public education, and evidence on the safety of ketamine after long-term use. They felt that monitoring was required to provide evidence for ketamine's safe use and administration, but there were concerns about the misuse of this information. Practical aspects included discussions about treatment duration, administration and accessibility (for example who would receive it, under what criteria and how).

**Conclusions:**

Patients are enthusiastic about ketamine treatment but need more information before national roll-out. The wider societal impact of ketamine treatment also needs to be considered and patients need to be part of any future roll-out to ensure its success.

## Background

Depression is the leading cause of mental health-related disorders^1^ and is projected to be the leading cause of global disease burden by 2030.^2^ Although treatment options exist, only one-third of those adequately treated reach remission. Some of this group have treatment-resistant depression (TRD), defined as being non-responsive to treatment after trialling at least two antidepressants.^3,4^ The poor long-term outcomes of people with TRD signals an urgent need for new treatment options.^3,5^

One promising treatment is the *N*-methyl-d-aspartate receptor antagonist, ketamine.^6,7^ Clinical trials and meta-analyses report a rapid antidepressant effect lasting 1–2 weeks following intravenous ketamine administration.^6–10^ In 2019, the esketamine nasal spray was approved as a treatment for adults with TRD in conjunction with oral antidepressants by both the US Food and Drug Administration and the European Medicines Agency.^11,12^ Despite this, the National Institute for Health and Care Excellence (NICE) recently made a preliminary decision against its recommendation for several reasons including its cost-effectiveness and concerns over stopping the treatment.^13^

Positive experiences of ketamine for treating depression have been reported by patients, carers and advocates^14,15^ but more information is required prior to its widespread roll-out as it poses challenges that come alongside the benefits. These include the potential for misuse, dependence, tolerance, withdrawal symptoms and physiological side-effects (for example urological/bladder problems),^16–18^ as well as the potential for illegal diversion to street markets or illegal access if a prescription is refused.^18,19^

## Patient views

In a previous study by our group, ten themes were raised by patients, carers and advocates with experience of ketamine as a treatment for TRD or used recreationally.^15^ The themes included better evidence on safety as a long-term treatment, the importance of monitoring, fear of data misuse, practical issues, cost and side-effects. Although that study included a range of participants, there was no specific information from those most likely to be prescribed ketamine (i.e. those with a diagnosis of depression with no previous experience of using it). This study fills this gap by providing an in-depth exploration of the attitudes of these people with lived experience of depression but no prior ketamine use, about ketamine as an antidepressant. We took an inductive approach in order to generate new themes with this novel group of participants; the views of this group are important to inform policy and practical decisions on its clinical use.

## Method

### Design

This qualitative study was in two stages: focus groups followed by member-checking validation sessions. The focus groups collected participants’ views and opinions of ketamine as a treatment for depression, their thoughts on prescribing and monitoring, and whether they had any concerns.

### Participants and recruitment

Participants were included if they were:

- at least 18 years old;
- had a depression diagnosis (self-reported or clinically confirmed experienced on two or more occasions); and
- had not used ketamine for treating depression.

They were excluded if they had a history of a psychotic or schizoaffective disorder, substance misuse or had received secondary care support for substance misuse within the past 6 months as these are contraindications for ketamine treatment.^18^

They were recruited via three channels: (a) posters at a local National Health Service (NHS) hospital; (b) a previous research study; and (c) three local service-user advisory groups. Recruitment stopped when no new themes emerged.

### Procedure

The study was granted NHS ethical approval from the South West – Frenchay Research Ethics Committee (18/SW/0232). Three focus groups and three member-checking sessions were held between 29 January 2019 and 30 July 2019.

Participants were asked for details of their history prior to being accepted to attend the focus groups and were remunerated for their time at each focus group, as well as travel expenses. Each focus group lasted between 60 and 90 min and was facilitated by two service-user researchers, using a topic guide based on the results of a public consultation day^15^ (Appendix). To ensure a shared understanding, a summary of ketamine treatment for depression was provided (supplementary data, section 1 available at https://doi.org/10.1192/bjo.2020.165). All groups were audio-recorded and brief notes taken. A summary of the extracted themes was provided to participants during a member-checking focus group 2 weeks later.

### Analysis

All discussions were transcribed by a service-user researcher who supported the focus groups, and themes were extracted using NVivo 12. Preliminary analyses identified broad themes and these analyses were presented in the member-checking sessions for validation. The final thematic analysis^20^ was carried out inductively by three researchers (C.O., S.M. and E.W.) using Pope et al's^21^ analysis framework which involves:

- familiarisation of raw data;
- identifying a thematic framework;
- indexing – applying the thematic framework to all the data by annotating the transcripts;
- charting – rearranging the data according to thematic framework;
- mapping and interpretation – defining concepts, mapping the range and nature of phenomena, and creating typologies.

A consensus was reached for the final codes, and the framework generated. Further information on the analytic process can be found in the Supplementary data, section 2.

## Results

Fourteen participants took part in the focus groups (group 1, *n* = 5; group 2, *n* = 4; group 3, *n* = 5; see Table 1). Only one participant was not able to attend the member-checking session.

**Table 1:** Participant characteristics

Characteristics	Participants (*n* = 14)
Female, *n* (%)	10 (71)
Age, years: mean (s.d.)	55.8 (13.6)
Years living with depression, mean (s.d.)	21.8 (13.8)^a^
Ethnicity, *n* (%)	
White British	8 (57)
Black/Black British	3 (21)
Other White background	2 (14)
Mixed ethnicity	1 (7)
Recruitment source, *n* (%)	
Poster advertisement	1 (7)
Previous study on depression	7 (50)
Local service-user advisory groups	6 (43)

a. *n* = 12, with missing data.

**Fig. 1:**
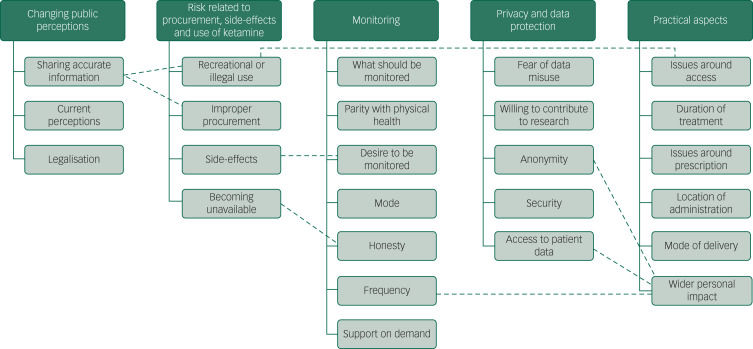
The five key themes and the subthemes emerging from thematic analysis of the focus group data.The dashed lines show where subthemes were linked.

Five key themes were identified (Fig. 1). Focus groups were run until no new themes appeared. All of the themes appeared in the first two focus groups and in the final group, no new themes emerged (Supplementary Table 1).

### Changing public perceptions of ketamine

Participants described ketamine as having a ‘reputation’ as a horse tranquiliser, anaesthetic and party drug, with potential side-effects and a perceived link to serious mental illness and death. These perceptions were formed through news stories, television shows, personal experience or friends taking it for recreational purposes.

Participants suggested ways to create a positive public perception of ketamine treatment was to emphasise sharing accurate information with the public on how it helps depression, and to differentiate the repurposed medical drug from the illegal versions, combined with information about its strict regulation and monitoring by doctors. They spoke about the importance of explaining any risks by putting this in context, such as explaining how the medical ketamine would be a much smaller dose and higher purity than that sourced illegally.

Several discussed how to differentiate ketamine from a party drug by giving it a different name. For example, one participant said:

‘And if- if- if how it's marketed creates that kind of perception of a division, or a distinction between ketamine and esketamine, that it's a different, slightly different, substance – it's safer, it's regulated – that will help.’ (Participant 1, Group 2)

Another argued that this did not disseminate accurate information, which was shared by others:

‘We're trying to hide the fact that it's ketamine and [also saying] it's ketamine and it's great for you.’ (Participant 4, Group 3)

Participants were worried about stigma. For example:

‘Yes, and also some people don't – even in my life, I haven't told everyone about being on antidepressants cos there's still that stigma. [murmurs of agreement in group] I can't imagine what it must be like for someone taking ketamine. Some people might really have a problem with that.’ (Participant 4, Group 1)

Participants also highlighted that the public perception may become more positive if ketamine was de-criminalised and therefore legally accessible.

### Risk related to procurement, side-effects and use of ketamine

Participants hypothesised that self-medication might drive a person to purchase ketamine illegally because they might ‘try anything’ if they are ‘desperate’ or ‘low and nothing is helping’, particularly if they cannot access ketamine through the NHS or it is too expensive privately. One participant said:

‘If somebody said you to “eat that glass it's gonna help you”, you'd do it. I mean, if you're that desperate you'd try anything and that would be my theory about ketamine if it becomes public knowledge that it can help depression.’ (Participant 5, Group 1)

Participants thought that criminals might ‘fake depression’, to procure the drug from NHS services and then sell it for profit, but this was not a great concern. Participants expressed a fear of ketamine being withdrawn because of perceptions of psychological or physical dependency or addiction, or a change in clinical guidelines. There might be dishonest symptom reporting, because of fear that it would be removed from their treatment plan. This problem might be lessened with evidence of the likelihood of tolerance or addiction to ketamine.

Although some disliked the idea of becoming ‘reliant’ on a medication, others compared it to taking a long-term medication for a physical health condition (for example for thyroid problems or managing diabetes). Some expressed concern that a rapid change in mood from ketamine's fast-acting antidepressant effect, could be a ‘jarring’ experience. A sudden improvement in functioning could risk overlooking environmental influences of depression, such as the home environment or wider socioeconomic inequalities, where ketamine treatment becomes ‘like putting a sticking plaster over… a massive problem’. Finally, side-effects were discussed, including adverse reactions from clinical trials. Participants wanted further scientific evidence to investigate side-effects.

### Monitoring ketamine use

Participants advocated strict monitoring with a preference for regular clinical reviews, especially as ketamine may have unknown side-effects. Monitoring was considered as personally beneficial alongside self-monitoring. Both patients and their families would gain reassurance from clinical feedback:

‘I would welcome it, personally. Because it's quite a serious drug and I think I would like to know there's someone at the other end who is following my progress and knowing what's going on.’ (Participant 4, Group 3)

Specific monitoring, including ketamine use, quality of life and mood was suggested with frequency depending on what was being monitored. Many valued regular monitoring at the start, ranging from once to multiple times a week, eventually decreasing the frequency as a person acclimatises to the treatment. Fluctuations in mood could be recorded more regularly, using a single question or tick box. Some participants wanted the mode of monitoring to be face-to-face assessment, with one preferring ‘anything but digital’. Others suggested text, phone call, online or a mobile app as more acceptable, with cybersecurity flagged as a potential issue. Less variable measures such as quality of life would be better assessed once a month, as more frequent checks could prove emotionally difficult. Despite this, participants were aware that this level of monitoring would be challenging as ‘the NHS is already stretched’. Many highlighted the lack of current antidepressant monitoring and difficulty accessing general practitioners (GPs) and mental health services. Participants were more inclined to be honest about their thoughts, cravings and tolerance to ketamine, if clinicians remain transparent about how this could affect their treatment. Support on demand would provide reassurance, like systems currently in place for monitoring physical health conditions:

‘I think it gives you a reassurance. I mean it comes back to the diabetes. If somebody's monitoring you and you know you've got a real person to pick up the phone to if there's something particularly wrong.’ (Participant 5, Group 1)

### Privacy and data protection around ketamine treatment

Despite a strong willingness to be monitored, participants were fearful of their data being misused and non-NHS organisations having access to clinical information:

‘I know from various types of independent sector organisations involved in healthcare, that, very often, the profit does dominate and I'm not saying that's true of all, but there is that potential for misuse of data… If it's just for healthcare or for research within, you know, the NHS and universities… you know, you sort of instinctively trust the integrity of those organisations more.’ (Participant 1, Group 2)

Participants wanted to know whether employers could have access leading to potential negative consequences. This was linked to discussions on stigma. Participants had varying views on acceptable access to ketamine treatment data and the scale of data being shared. Some argued that automatic access should be given only to their immediate clinical team, whereas others expressed trust in the NHS and were open to ‘blanket’ NHS access ‘to monitor how well or otherwise this treatment's working’. The type of information monitored depended on who could access the data, but there was a strong willingness to share ‘as much data as possible’ to contribute to research on ketamine treatment:

‘If it's going to help other people and they can gather enough evidence to make a change, I would really welcome it.’ (Participant 4, Group 3)

Overall, participants agreed that personal choice by giving explicit consent or having the ability to opt out of data sharing beyond their care team were the best options. When questioned, participants were willing for data relating to their treatment to be shared, if processes are clearly explained, monitoring is confidential, data is anonymised and shared only via secure systems.

### Practical aspects of ketamine use

Participants wondered about the duration of treatment, particularly whether it is a one-off medication to reduce suicidal ideation or for long-term use to manage depression, and if it would be used in conjunction with other therapies. Participants discussed their experiences of using antidepressant medications, saying it is often trial-and-error, and sometimes a long process. Many liked the idea of ketamine being fast-acting so they can resume ‘normal’ life:

‘So, when people ask me “what do you want?”…You want to be able to say, “If I take this now, I can go back to my life and I want it now”.’ (Participant 5, Group 1)

Participants wondered whether ketamine would only be prescribed by specialists or if it could be obtained from a GP, and whether it would be on repeat prescription without review. For reasons of safety, many felt a specialist clinic may be the best location for administering the drug, compared with a GP surgery or at home:

‘I think it would be better to, um, to be somewhere with someone who understands depression and mental illness, rather than an overworked GP… – I mean I'm lucky if I see the same GP twice at my practice –… So, that also makes things more difficult because you can't build up a relationship with the doctor on something like this because it's never the same one. So, I think, yeah, a clinic or something like that would be better.’ (Participant 1, Group 3)

Some suggested allowing GP prescription only once depression is controlled. Finally, they liked the idea of having choice in the form of delivery, particularly for individuals who may struggle with swallowing or taking it intravenously.

They thought ketamine should be accessible on the NHS when approved but some felt that clinicians may be unwilling to prescribe ketamine despite approval if it is more expensive than cheaper, conventional treatment options. This was based on previous experience with other medications. There was apprehension that minimising costs could be prioritised over well-being, due to a lack of NHS funding and high prices set by pharmaceutical companies.

Important considerations were raised about the wider impact of ketamine treatment on day-to-day life, such as taking time off from work, travelling to the clinic when feeling unwell and the burden on carers. Some wanted to know whether ketamine could affect other treatments and conditions, including intellectual disabilities. All groups noted that disclosing ketamine treatment could increase the cost of personal insurance policies (for example life, travel, health). Participants queried who would have access to ketamine, as the definition of TRD could apply to many:

‘But don't you think probably everybody who's depressed are treatment-resistant – cos I'm sure everybody's been on more than two different anti- antidepressants.’ (Participant 3, Group 2)

As a result, participants stressed that it not only needs to be available for those experiencing suicidal thoughts, but the ‘debilitating’ nature of chronic depression should warrant this treatment option even in the absence of suicidality.

## Discussion

### Main findings

To the best of our knowledge, this is the first study to conduct an in-depth exploration of patients’ views about ketamine as a potential treatment for depression, with service-user researchers involved at each stage of the process. Some themes overlapped with those from Jilka et al^15^ but we also found new challenges that patients were concerned about, including the immediate side-effects, practical considerations of ketamine treatment, and the effect of a rapid change of mood. Ketamine was enthusiastically welcomed as a potential new treatment, but discussions featured important considerations. Conversations around personal preference and patient values highlighted the importance of taking a person-centred approach throughout the care process especially as patient preference may predict treatment response.^22^ Care teams may also want to consider family and friends who play important roles in supporting recovery.

Participants showed a real curiosity about how ketamine and antidepressants work in the brain, a desire for more research and a willingness to take part in that research. Normalising ketamine as a treatment is vital,^15^ and patients in our study highlighted that changing current public perceptions is key. This may alleviate worries about ketamine being a ‘party drug’, and a great deal of discussion concerned how best to differentiate medical ketamine from its murkier reputation. For example, by emphasising the differences between medical ketamine and the recreational form, with clinical trials using a low dose to reduce the risk of dependency, addiction or adverse physical reactions.^19,23^ Patients felt that the risk of ketamine being diverted from clinics to street markets should be considered secondary to its health benefits, reflecting clinician opinion.^24^ However, if factors such as cost affect its accessibility and availability through the NHS, the likelihood of self-medication through recreational use should be addressed.^14,25^ Changing the perception of ketamine and educating the public on this important distinction could minimise this risk.

Participants felt that ketamine treatment should be considered in conjunction with addressing psychosocial factors that may contribute towards a person's depression, with some participants preferring non-pharmacological approaches such as talking therapy. The importance of self-care strategies was emphasised, and there was more discussion about the influence of the environment and inequalities than in a previous study.^15^ Although being fast-acting was a benefit, some expressed caution, as rapid mood changes may be unsettling or mask wider problems in a person's life. This finding is in line with Singh et al^19^ and highlights patients’ desire for careful clinical management of the rapid, short-term improvement in depressive symptoms and the potential for relapse that may follow.

The importance of strict expert clinical monitoring was shared by all and self-administration was cautioned, reflecting concerns of potential misuse if a patient has access to high doses.^19^ There was much discussion about having a specialist community clinic rather than visiting a GP. This would also favour the implementation of a comprehensive, flexible monitoring system – an essential component of overseeing ketamine use^24^ to mitigate patient concerns about unknown side-effects and developing tolerance. Fears of data misuse led to a desire for some level of control over how patient data may be shared, although there was an overriding willingness to have anonymised data contribute to the ketamine evidence base.

### Comparison with findings from research on health professionals’ opinions

Our patient-focused work builds on previous research on mood disorder experts’ (such as clinicians, academics and researchers) opinions on ketamine treatment.^26^ They outlined their limited confidence in the efficacy of ketamine treatment and called for more research; our participants requested the same, in a lay and accessible form, particularly on side-effects. The issue of side-effects was highlighted by mood disorder experts on discussions around tolerability and potential for misuse. They outlined the importance that side-effects are fully understood before widespread clinical use and our participants corroborated their views. But they expanded issues of tolerability and misuse to the idea of ‘faking depression’ to procure ketamine, and whether tolerability would mean ketamine is withdrawn because of dependency or addiction. Our discussions around misuse also spanned data privacy and security. The mood disorder experts called for ketamine's administration to be both simple and cost-effective. The current method of intravenous infusion is possible in specialist units, but may pose problems in routine clinical practice. Our conversations on administration and settings were centred around personal preferences (for example, having a choice in the form of ketamine delivery).

Mood disorder experts recommended that future research should prioritise the optimal course and duration of acute ketamine therapy for those who show a robust response initially. Patients supported this, but emphasised that the patient voice should be central to any future discussion of ketamine treatment. This is important because our participants highlighted new issues that will need to be considered to ensure uptake and adherence.

### Strengths and limitations

This study places the patient voice at the heart of the research, evidencing participatory research and direct collaboration; both fundamental elements of translational research,^27^ with service-user researchers and member-checking to ensure participant validation. The social and cultural diversity provided a mix of perspectives and attitudes and encouraged a rich discussion across topics, and the participants were typical of those who could be offered ketamine treatment but different to our previous work.^15^ Although the sample reflected the demographic balance of patients present within South London services, the results need further validation with men and those from an Asian background. Participants were on average aged 56 so further research is needed with younger people who may be offered ketamine for TRD in the future.

Our sample of patients were mainly recruited from another study or advisory groups, and therefore may be better-informed about research. Future studies may wish to consider populations less involved in research for further generalisation.

### Implications

The varying effectiveness of current antidepressants and psychological therapies means that the prospect of new treatment options such as ketamine are highly anticipated by patients and clinicians. But success will be based on a person-centred approach, where patients access a choice of treatment options, an NHS guiding principle.^28^

When ketamine treatment is available in the NHS, the next hurdle will lie in how accessible it is to those who need it, and which criteria must be met before it can be offered. Many individuals with depression remain under the care of primary care services, and patients noted that access to ketamine could therefore be dependent on how informed their GP is on ketamine as a viable treatment option, and their ability to provide the appropriate referrals. Training for primary care clinicians would be required to ensure consistency in care.

Patients value a clear understanding of the existing research about ketamine, the distinction between the medical version and the street drug, and its efficacy in the treatment of depression. With research in this area growing quickly, patients called for additional evidence on the mechanisms of ketamine, its side-effects, the longer-term impact and how it could form part of a treatment plan. Future work should consider a deductive approach to test specific hypotheses on patient views. This is particularly important as NICE's decision to not recommend ketamine was partly because there was not yet enough long-term evidence to support the use of nasal esketamine alongside another antidepressant.^13^

In conclusion, this study identified further considerations and solutions to the challenges of ketamine treatment for depression. Challenges ranged from the stigmatising effect of public perceptions to preferences for treatment delivery. There is a pressing need to embed patients’ preferences early in the development of the ketamine treatment pathway and to promote the patient voice as a central part of future roll-out. This would ensure a novel treatment pathway that is acceptable, feasible, cost-effective and fit-for-purpose.

## Data Availability

The data that support the findings of this study are available from the corresponding author, S.J., upon reasonable request.

## Supplementary material

For supplementary material accompanying this paper visit http://dx.doi.org/10.1192/bjo.2020.165. click here to view supplementary material

## Appendix

### Focus group topic guide

**Table:**

Topic	Content
**Introduction**	
	Researchers to introduce themselves and the project.
	Introduce audio-recording equipment, how long the session will last and breaks.
	Explain consent procedure: go through information sheet.
	Explain confidentiality (including use of quotes) and agree ground rules: (a) keep phones off or on silent, if you get a phone call you have to answer the reception area just outside is available; (b) location of toilets; (c) respect each other's point of views, for example waiting for others to finish what they want to say before sharing your point; (d) anything that is mentioned during the group discussion, for example personal information or experiences, should not be shared outside the group (e) does anyone in the group want to add anything further to the ground rules?
**Ice breaker**	
	Introduce yourself and say where you have come from (or something similar).
	Introduce brief summary on ketamine use in depression, and how this focus group is following on from the patient consultation day.
**Main discussion**	
Aim: to understand the behaviour and attitudes of patients who are considering ketamine as an antidepressant option to help inform policy and practical decisions about its clinical use	
	What aspects of an antidepressant are important to you?
	What are your perceptions of the risks and benefits of being prescribed an antidepressant which worked rapidly – within a day?
	Some people at the consultation day thought that others may try to access ketamine illegally.… Do you think people will access ketamine illegally as a treatment for depression? (a) What do you think the drawbacks would be?
	*Suggest 5 min break*
Aim: to understand patient views about personal data collection and monitoring	
	What are your views on the monitoring of your personal data in medical records? (a) Would this differ if you could see your record and could control who else could see it?
	How would you feel if this information included ketamine use, your quality of life and your mood in order to be prescribed ketamine? (a) How often would you be prepared to provide this information?
	How would you feel about having to supply information about how much you were thinking about, craving and/or becoming ‘tolerant’ to ketamine in order to be prescribed ketamine?
	Would you like to add anything else?
**Close**	
	Explain how data will be used.
	Remind participants about confidentiality.
	Explain the next steps in the project, i.e. we will carry out thematic analysis of the focus groups – invite participants to review themes once defined to ensure their views are reflected in the analysis.
