# Supplementary material for: Ketamine treatment for depression: qualitative study exploring patient views
Source: BJPsych Open. 2021 Jan 11;7(1):e32. doi: 10.1192/bjo.2020.165 (PMC8058884; doi:10.1192/bjo.2020.165)
Supplement: Supplementary file 1 [file S2056472420001659sup001.docx]

Supplementary Information.

CONTENTS

Summary sheet about ketamine for treatment-resistance depression…… Page 2

Additional information on focus group analysis……………………………………….Page 3

Supplementary Table 1…………………………………………………………………………...Page 4

1. **Brief summary of information about ketamine for treatment-resistant depression given to participants in the focus groups.**

**Research**

- There is strong evidence that ketamine is an effective antidepressant.
- There is also growing evidence that it can reduce suicidal thoughts.
- There is some evidence that these effects are maintained through regular and longer-term prescribed-use of ketamine, initially twice-weekly then up to fortnightly.

**Situation**

- The use of ketamine for the treatment of depression is relatively new, so the best way of taking it has not been fully worked out yet.
- Currently, there is little UK experience of using ketamine as an anti-depressant. Experience is concentrated in Oxford, Northampton and Middlesbrough.
- Ketamine is being used for people with treatment resistant depression, but almost exclusively in private clinics in the UK.
- It is recommended that people considering ketamine as a treatment, and their carers, should be provided with clear information, including on the current evidence and potential risks.

**Potential problems**

- Ketamine is used as an illegal party drug. Side effects which occur with abuse may possibly also occur with frequent or long term medical use.
- Tolerance to ketamine can sometimes occur (this means the person no longer responds to prescribed ketamine in the way they initially did).
- Physical dependence on ketamine occurs when levels of the drug used are much higher than in medical use. Psychological dependence is occasionally seen when used medically.
- A survey has suggested that patients may choose to self-medicate with illegally supplied ketamine if access to medically controlled ketamine is difficult.
- There is a concern that private prescriptions might encourage ‘doctor hopping’ in those who become dependent on ketamine

1. **Supplementary information: Methods - additional information on focus group analysis**

Three researchers independently read the transcripts of each focus group to identify themes in order to construct a thematic framework. The researchers discussed the themes to agree a final framework. We followed Pope et al’s (2000) analysis framework which involves: (1) Familiarisation of raw data; (2) Identifying a thematic framework—identifying all the key issues and concepts which produces a detailed index of the data, which labels the data into manageable chunks for subsequent retrieval and exploration; (3) Indexing—applying the thematic framework or index systematically to all the data in textual form by annotating the transcripts with numerical codes from the index; (4) Charting—rearranging the data according to the appropriate part of the thematic framework to which they relate; (5) Mapping and interpretation—using step 4 to define concepts, map the range and nature of phenomena, create typologies and find associations between themes with a view to providing explanations for the findings. A consensus was reached for the final codes, and the framework generated.

Our methodology ensured trustworthiness of the final themes through a member checking process. Ahead of this member checking process, a researcher who had been present at/led the initial focus group produced a summary of the broad themes/sub-themes that came up in the focus group. This summary was produced by re-listening to the audio-recording, using notes taken during the group, and through discussion between the researchers. At the member checking focus groups, participants were presented with and went through this summary theme by theme. Participants expressed their opinions, agreed, clarified and disagreements with the presented summary. New insights and nuances were developed by this process. Data from all member checking transcriptions were also coded into the framework, and the new insights used to accurately describe the final themes.

Throughout this whole process, researcher perspectives were considered according to the principles of reflexivity. Each focus group and member checking focus group was led by a researcher with training in running focus groups and leading mental health service user groups. Two women, and one man (the lead author) facilitated the focus groups. The final in-depth coding were conducted independently by one focus group facilitator (CO), and two qualitative researchers who were not present in the focus group to help minimise bias (SM and EM). All researchers had a background in mental health research and/or clinical psychology, and none were directly involved in the clinical care of the participants.

Focus groups were run until no new themes appeared. All of the themes appeared in the first two focus groups and in the final group, no new themes emerged.

Reference

Pope, C, Ziebland, S, Mays, N. Analysing qualitative data. Br Med J 2000; 320: 114

**Supplementary Table 1. Key themes, subthemes and sub-subthemes extracted from the thematic analysis of the focus groups**

| **Theme** | **Subtheme** | **Group 1^a^** | **Group 2 ^a^** | **Group 3 ^a^** |
| --- | --- | --- | --- | --- |
| Changing public perceptions of ketamine | ***Current perceptions*** *of ketamine* | ✓ | ✓ | ✓ |
|  | *Importance of* ***sharing accurate information*** *with public and patients* | ✓ | ✓ | ✓ |
|  | 1. ***Ways to communicate*** | ✓ | ✓ | ✓ |
|  | 1. ***Rigorous testing and monitoring*** | ✓ | ✓ | ✓ |
|  | 1. ***Repurposing drugs*** *for medicinal value* | ✓ | ✓ | ✓ |
|  | 1. ***Differentiating from the ‘party drug’*** | ✓ | ✓ | ✓ |
|  | ***Legalisation*** *of ketamine* | × | ✓ | ✓ |
| **Risks of ketamine** | ***Recreational or illegal use*** | ✓ | ✓ | ✓ |
|  | *Risk of* ***people without depression procuring ketamine*** *on the NHS* | ✓ | ✓ | ✓ |
|  | *Fear of* ***wanting to continue ketamine*** ***treatment and*** ***it becoming unavailable*** | × | ✓ | ✓ |
|  | ***Side effects:*** | ✓ | ✓ | ✓ |
|  | 1. *Potential for* ***addiction*** *or* ***dependence*** | ✓ | ✓ | ✓ |
|  | 1. ***Tolerance*** *and* ***increased use*** | ✓ | ✓ | ✓ |
| **Monitoring of ketamine use** | ***Desire to be monitored*** | ✓ | ✓ | ✓ |
|  | 1. ***Personal benefits*** *of monitoring* | ✓ | ✓ | ✓ |
|  | ***What*** ***should be monitored*** | ✓ | ✓ | ✓ |
|  | ***Frequency*** *of monitoring* | ✓ | ✓ | ✓ |
|  | ***Mode*** *of monitoring* | ✓ | ✓ | ✓ |
|  | ***Honesty*** *in monitoring* | × | ✓ | ✓ |
|  | *Availability of* ***support on demand*** | ✓ | ✓ | ✓ |
|  | ***Achieving parity*** *with physical health conditions* | ✓ | × | ✓ |
| **Privacy and data protection around ketamine treatment** | *Fear of* ***data misuse*** | ✓ | ✓ | ✓ |
|  | ***Access*** *to patient data:* |  | | |
|  | 1. ***Employer access*** | ✓ | ✓ | ✓ |
|  | 1. ***Acceptable data sharing and access:*** |  | | |
|  | - 1. ***Non-NHS*** *access* | ✓ | ✓ | ✓ |
|  | - 1. ***Scale of data sharing*** | ✓ | ✓ | ✓ |
|  | - 1. ***What data is acceptable to share*** | ✓ | ✓ | ✓ |
|  | - 1. ***Personal choice and consent*** | ✓ | ✓ | ✓ |
|  | *Willingness to* ***contribute to research*** | ✓ | ✓ | ✓ |
|  | ***Anonymity*** | ✓ | ✓ | ✓ |
|  | ***Security*** | ✓ | ✓ | ✓ |
| Practical aspects of ketamine treatment | ***Duration*** *of treatment* | × | ✓ | ✓ |
|  | *Issues around* ***prescription*** | ✓ | ✓ | ✓ |
|  | ***Location*** *of administration* | ✓ | ✓ | ✓ |
|  | 1. *Involvement of* ***research bodies*** | ✓ | ✓ | ✓ |
|  | ***Mode of delivery*** | ✓ | ✓ | ✓ |
|  | ***Issues around access:*** |  | | |
|  | 1. ***Cost*** | ✓ | ✓ | ✓ |
|  | 1. ***Who ketamine should be for*** | ✓ | ✓ | ✓ |
|  | ***Wider personal impact*** *of taking ketamine* | ✓ | ✓ | ✓ |
| **Note**. **^a^** A ‘tick’ indicates whether this theme was discussed in the respective focus group. This was used to calculate data saturation. | | | | |
